# A selective C5a-derived peptidomimetic enhances IgG response following inactivated SARS-CoV-2 immunization and confers rapid disease resolution following murine coronavirus infection

**DOI:** 10.3389/fimmu.2025.1470034

**Published:** 2025-04-04

**Authors:** Andrew J. Neville, Mackenzie E. Conrin, Thomas T. Schulze, Paul H. Davis

**Affiliations:** ^1^ Department of Biology, University of Nebraska at Omaha, Omaha, NE, United States; ^2^ Department of Pathology, Microbiology, and Immunology, University of Nebraska Medical Center, Omaha, NE, United States

**Keywords:** coronavirus, MHV, peptide therapeutics, adjuvant, EP67, complement pathway, C5a, SARS-CoV-2

## Abstract

The host complement system is a critical component of innate immunity and serves as a principal mechanism of pathogen defense in mammals. EP67 is an engineered decapeptide derived from the C terminus of human complement protein C5a, which displays selective immunostimulatory activity. EP67 preferentially activates phagocyte mononuclear cells but shows minimal activity towards inflammatory granulocytes, including neutrophils. Previous studies of viral infection showed that EP67 possessed antiviral efficacy when used following infection and enhanced antibody responses to antigen challenges when used as an adjuvant. Here, we show in a rodent model that immunization with inactivated γ-irradiated SARS-CoV-2 in combination with EP67 can produce elevated nucleocapsid-specific IgG antibodies compared to viral lysate alone, supporting an enhanced adaptive immune response. Additionally, intranasal administration of EP67 following infection with live MHV-A59 coronavirus resulted in a rapid health improvement in symptomatic infections compared to PBS vehicle controls. Taken together, these results suggest EP67 shows efficacy towards betacoronaviruses when used as an adjuvant during immunization or as a therapeutic during active infections. Moreover, these findings continue to support the capability of EP67 as an antiviral agent and a useful immunostimulatory peptide.

## Introduction

The complement system is an innate immune mechanism largely conserved across mammalian species and acts against infectious agents. Components of the complement system work in an orchestrated manner to subdue pathogens. This is achieved in myriad ways, including the deposition on the target pathogen, which leads to membrane disruption or opsonization, and the recruitment of immune cells to the area of activity through complement mediators. One such mediator is the complement subunit C5a, which is produced via proteolysis of the parent C5 molecule. Human C5a is 74 amino acids in length and acts in a chemotactic manner to recruit and activate immune cells bearing the C5a receptor (1). The native C5a molecule potently induces recruitment of phagocytes, including macrophages and monocytes, and less so inflammatory granulocytes, such as neutrophils and mast cells (2). Due to its ability to recruit, activate, and induce degranulation in the latter, full-length C5a is appropriately known for its inflammatory effects.

The C5aR1/CD88 interactive peptide, EP67, is a small decapeptide derived from the C terminus of human C5a with the sequence YSFKDMP[MeL]aR where “MeL” is N-methyl leucine and “a” is D-alanine. It was designed to act as a non-inflammatory immunostimulant and immune adjuvant by selectively recruiting and activating phagocytic antigen-presenting cells whilst reducing inflammatory effects that can occur with the recruitment of granulocytic cells (3–5). EP67 has demonstrated effectiveness in murine models of viral infection, such as protection against cytomegalovirus (CMV) infection via adjuvant activity (2). The antiviral activity may be partially due to EP67’s ability to promote T_H_1 immune activation via the C5aR1/CD88 receptor pathway (5). Additionally, EP67 has demonstrated efficacy as additional in bacterial and viral infections (6–8). Therefore, we posited that EP67 could work in a therapeutic manner against a potent, symptomatic coronavirus infection.

In 2020, the worldwide spread of SARS-CoV-2, a betacoronavirus, led to a pandemic with high morbidity and mortality. With the relative paucity of broad-spectrum antiviral agents, vaccine approaches were primarily employed to reduce spread and attenuate the more severe effects resulting from viral infection. Several animal models exist for modeling coronavirus infections; however, access barriers such as cost, availability, and housing requirements (e.g. BSL-3 facilities) can render these impractical for many laboratories (9). The betacoronavirus, Murine Hepatitis Virus (MHV), naturally infects mice and various disease pathologies of human SARS-CoV and SARS-CoV-2 infections can be modeled *in vivo* with varied animal and MHV strains (10–12). Specifically, C57BL/6 mice inoculated intranasally with MHV strain A59 (MHV-A59) have shown acute pneumonia and lung pathology due to viral spread in the lungs and leukocyte infiltration beginning as early as 2 days post-infection (2 dpi) (12). Using this model, we administered a single dose of EP67 intranasally after the onset of symptoms (3 days post-infection; 3 dpi) to evaluate the ability of EP67 treatment to alleviate severe health decline in infected mice. Mice receiving EP67 recovered quickly compared to untreated infected mice who significantly declined in health. This suggests that EP67 could serve as an effective antiviral therapeutic against the betacoronavirus MHV. Additionally, we show that EP67 increased long-term plasma IgG nucleocapsid antibodies when coadministered with inactivated SARS-CoV-2, with a similar increase in spike protein IgG. Together, these findings further support the antiviral efficacy of EP67 with potentially broader applications to additional viral diseases.

## Materials and methods

### Mouse hepatitis virus A59 propagation and preparation

17Cl-1 mouse fibroblasts and MHV-A59 viral stocks were graciously donated by Stanley Perlman, University of Iowa (13). Sanger sequencing confirmed the native MHV-A59 genotype. 17Cl-1 cells were cultured and used for MHV-A59 propagation, generation of high titer stocks, and quantification of plaque forming units (PFUs), as previously described (14). Briefly, 17Cl-1 cells were cultured in High-Glucose DMEM (Lonza BioWhittaker, cat# 12614F) supplemented with 4 mM L-alanyl-L-glutamine (Corning, cat# 25-015-CI), 1X penicillin-streptomycin (Hyclone, cat# SV30010), and 10% heat-inactivated fetal bovine serum (Gibco, cat# A3840001), further referred to as DME10. High-tier stocks were generated, plaque-purified, and stored at -80°C until use. Titers of viral stocks were quantified via plaque assays using 17Cl-1 cells inoculated with serially diluted viral stocks, followed by crystal violet staining to determine the number of plaques. PFU values were calculated as previously described (14).

### Plasma IgG response following EP67-adjuvanted inactivated SARS-CoV-2 immunization

8-week-old female Swiss Webster (CFW) mice were purchased from Charles River Laboratories (Wilmington, Massachusetts, USA), housed in ventilated cages with a 12-hour light/dark cycle, and given water and food *ad libitum* as approved by IACUC 20-058-03. 11-week-old female Swiss Webster (CFW) mice were anesthetized via isoflurane inhalation and received a 20 µL intranasal (IN) inoculation (10 µL/nostril). On Day 0, mice received a mixture of either PBS (control; n=6 mice) or 100 µg EP67 adjuvant (n=3 mice) and 2.66 x 10^3^ PFU of inactivated, γ-irradiated SARS-CoV-2, isolate USA-WA1/2020 infected Vero E6-heACE2 cell lysates (BEI Resources, cat# NR-53910). On Day 24, both groups of mice received the same inoculum as on Day 0. On Day 42, a 5 mm Goldenrod™ Animal Lancet (MEDIpoint, cat# GR-5MM) was used to puncture and collect blood via the submandibular bleeding method (15). Blood samples were collected directly into K_2_EDTA Microvette CB 300 capillary blood collection tubes (Starstedt, cat# 16.444.100). Samples were then centrifuged at room temperature at 2,000 x *g* for 6 minutes, and the plasma-containing supernatant was pipetted into 1.5 mL Protein LoBind tubes (Eppendorf, cat# 022431081) on ice. Plasma samples were immediately transferred and stored at -140°C until analysis. No hemolysis was observed in the plasma samples.

Enzyme-linked immunosorbent assays (ELISAs) were prepared using the Antigen-Down ELISA Development Kit (ImmunoChemistry Technologies, cat# 9101). All incubations were performed under light-protected conditions. Recombinant SARS-CoV-2 Nucleocapsid (N) protein (BEI Resources, cat# NR-53797, Lot# MF14JL0301; produced by Sino Biological, cat# 40588-V08B)) and Spike (S) glycoprotein (BEI Resources, cat# NR-55614, Lot# 70045340) were diluted to 2.0 µg/mL and 1.0 µg/mL, respectively, in 1X Antigen Coating Buffer (ImmunoChemistry Technologies, cat# 6247) and 50 µL was added per well in Nunc MaxiSorp flat-bottom 96-well plates (Thermo Scientific, cat# 44-2404-21), sealed with an adhesive plate sealer (R&D Systems, cat# DY992), and incubated at 4°C for 48 hours. Plates were equilibrated to room temperature, followed by aspiration of the antigen coating solution, and each well washed twice with 350 µL/wash of 1X ELISA Wash Buffer (ImmunoChemistry Technologies, cat# 651) using an ELx50 automated microplate washer (BioTek Instruments, cat# ELX50/8). 250 µL of General Block ELISA Blocking Buffer (ImmunoChemistry Technologies, cat# 632) was added per well, and the plate was sealed and blocked for 24 hours at room temperature, followed by an additional 16 hours at 4°C. Plates were equilibrated to room temperature before block buffer was aspirated. 50 µL of mouse plasma, pre-diluted 1:500 in General Serum Diluent (ImmunoChemistry Technologies, cat# 648), was added per well, plate sealed, and incubated for 8 hours at 4°C. A positive control anti-SARS-CoV-2 Nucleocapsid Antibody, Mouse IgG (Acro Biosystems, caat# NUN-S47A1), diluted in General Serum Diluent was used at 10.0 and 0.1 ng/mL. Rabbit IgG Polyclonal Anti-SARS-Related Coronavirus 2 Spike Glycoprotein (BEI Resources, cat# NR-52947) at 1:3000 and 1:30,000 dilutions in General Serum Diluent were used as the Spike glycoprotein positive control. Plates were returned to room temperature, samples aspirated, followed by five consecutive washes of each well using 350 µL/wash. 50 µL of Peroxidase-conjugated AffiniPure Goat Anti-Mouse IgG (H+L) (Jackson ImmunoResearch Labs, cat# 115-035-146; Lot# 158821) diluted 1:5,000 (160 ng/mL) in 1X Antigen-Down HRP Conjugate Stabilizer (ImmunoChemistry Technologies, cat# 6102) was added to each well. For the Rabbit IgG polyclonal anti-Spike glycoprotein positive control detection, a 1:10,000 dilution of 0.8 mg/mL Peroxidase-conjugated AffiniPure Mouse Anti-Rabbit IgG (H+L) (Jackson ImmunoResearch Labs, cat# 211-035-109) in General Serum Diluent. The plates were sealed, and incubated for 1 hour at room temperature., The plate was washed with seven consecutive washes using 350 µL per well, with the last wash including a 60-second soak before aspiration. 75 µL of TMB 1-Component HRP Microwell Substrate (ImmunoChemistry Technologies, cat# 6276) was added to each well and incubated for 10 minutes, followed by the addition of 75 µL of Stop Solution (ImmunoChemistry Technologies, cat# 6282) per well. Plates were gently tapped to ensure complete mixing, and absorbances were measured at 450 and 570 nm using a BioTek Synergy LX microplate reader (BioTek Instruments, cat# SLXFA). The corresponding absorbance measurements were reported as optical density (OD) values in the BioTek Gen5 software version 3.09 (BioTek Instruments, Winooski, Vermont, USA). OD_570_ nm readings were subtracted from the OD_450_ nm values to correct for optical imperfection background noise and to generate the corrected OD_450_ values used for statistical analyses. Each mouse plasma sample was assayed in technical duplicates. A two-tailed unpaired t-test for statistical significance was performed using GraphPad Prism version 10.4.1. Colored symbols represent the average corrected OD_450_ of two technical replicates measurements for each mouse, and the corrected OD_450_ group mean ± SEM are displayed as long and short bars, respectively. The background signal of the assay is displayed as a horizontal dotted line.

### MHV-A59 mouse infection and treatment

Male C57BL/6J mice at six weeks of age were purchased from Jackson Labs (Bar Harbor, Maine, USA), housed in ventilated cages with a 12-hour light/dark cycle, and given water and food *ad libitum* in accordance with IACUC 20-058-03. Mice acclimated for one week before beginning the study. Throughout the study, individual weights were recorded daily, and mice were inspected twice daily for signs of illness. On Day 0 (0 dpi), 7-week-old male C57BL/6J mice (*n*=10 per group) were exposed to isoflurane via the drop method and received an intranasal (IN) inoculation. Each mouse was administered a total volume of 20 µL (10 µL/nostril) using a p200 pipette equipped with a 200 µL tip. The inoculum consisted of either 1.1 x 10^6^ PFU of MHV-A59 mixed with 17-Cl1 cell lysate in DME10 for the two infected groups or 17Cl-1 lysate in DME10 as a mock infection for the uninfected control group.

On Day 3 post-infection (3 dpi), mice were anesthetized using isoflurane and IN administration of 20 µL (10 µL/nostril) of either 100 µg of EP67 (5 µg/µL) in sterile 1X phosphate-buffered saline (PBS) (Fisher BioReagents, cat# BP3994) or sterile 1X PBS only for the control group. On Day 5 post-infection, a random number generator was used to remove three mice from each group to preserve tissues. To account for any weight-related changes the EP67 treatment may have produced, the lysate mock-infected EP67-treated mice constituted the non-infected control group. Two-tailed unpaired t-tests were calculated using GraphPad Prism version 10.4.1 to determine statistical significance. Colored symbols represent individual mouse weights, and the group mean ± SEM are displayed as long and short bars, respectively.

Lungs were dissected from euthanized mice at two days post-treatment (5 days post-infection; *n*=3 per group) and seven days post-treatment (10 days post-infection; *n*=4 per group) and immediately placed in 3 mL of cold RNAlater™ Stabilization Solution (Invitrogen, cat# AM7020) and placed on ice. The mouse lungs in RNAlater solution were incubated overnight at 4°C. Lungs were thawed on ice, blotted with a Kimwipe to remove residual RNAlater solution, and the mass of each mouse lung was measured using an analytical balance.

### EP67 synthesis

Using HCl as the counter ion, EP67 was commercially synthesized and validated via electrospray ionization mass spectrometry by CPC Scientific Inc. (Sunnyvale, CA, USA; lot #: CQ-10-00782). Reversed-phase high-performance liquid chromatography (RP-HPLC) analysis determined a purity of 96.5%, while amino acid analysis determined the peptide content to be 88%.

### Murine macrophage exposure

RAW264.7 mouse macrophages (ATCC, cat# TIB-71) were grown in a humidified incubator at 37°C and 5% CO_2_, and cultured in High-Glucose DMEM (Lonza BioWhittaker, cat# 12614F) supplemented with 4 mM L-alanyl-L-glutamine (Corning, cat# 25-015-CI), 1X penicillin-streptomycin (Hyclone, cat# SV30010), and 10% heat-inactivated fetal bovine serum (Gibco, cat# A3840001). Cells were plated at 0.5 x 10^6^ cells/well (3 mL media/well) in 6-well cell culture-treated plates (Thermo Scientific, cat# 130184) and incubated for 24 hours to allow for cell attachment. Media was replaced with either fresh media only (control), or fresh media containing a final concentration of the following treatments: EP67 (50 µg/mL), recombinant human complement component C5a protein (100 ng/mL) (R&D Systems, cat# 2037-C5-025/CF), or lipopolysaccharide (LPS) (1 µg/mL) (Invitrogen, cat# 00-4976-93) and incubated for 16 hours. Each condition was tested in 3 separate wells (n=3 per condition). Supernatants were collected and secreted TNF-α protein levels were determined using the ELISA MAX™ Deluxe Set Mouse TNF-α Kit (BioLegend, cat# 430904) according to the manufacturer’s instructions. The supernatant from each well was assayed via ELISA in technical duplicates. A two-tailed unpaired t-test for statistical significance was performed using GraphPad Prism version 10.4.1. The group mean ± SEM of supernatant TNF-α levels are displayed.

## Results and discussion

**Figure 1:**
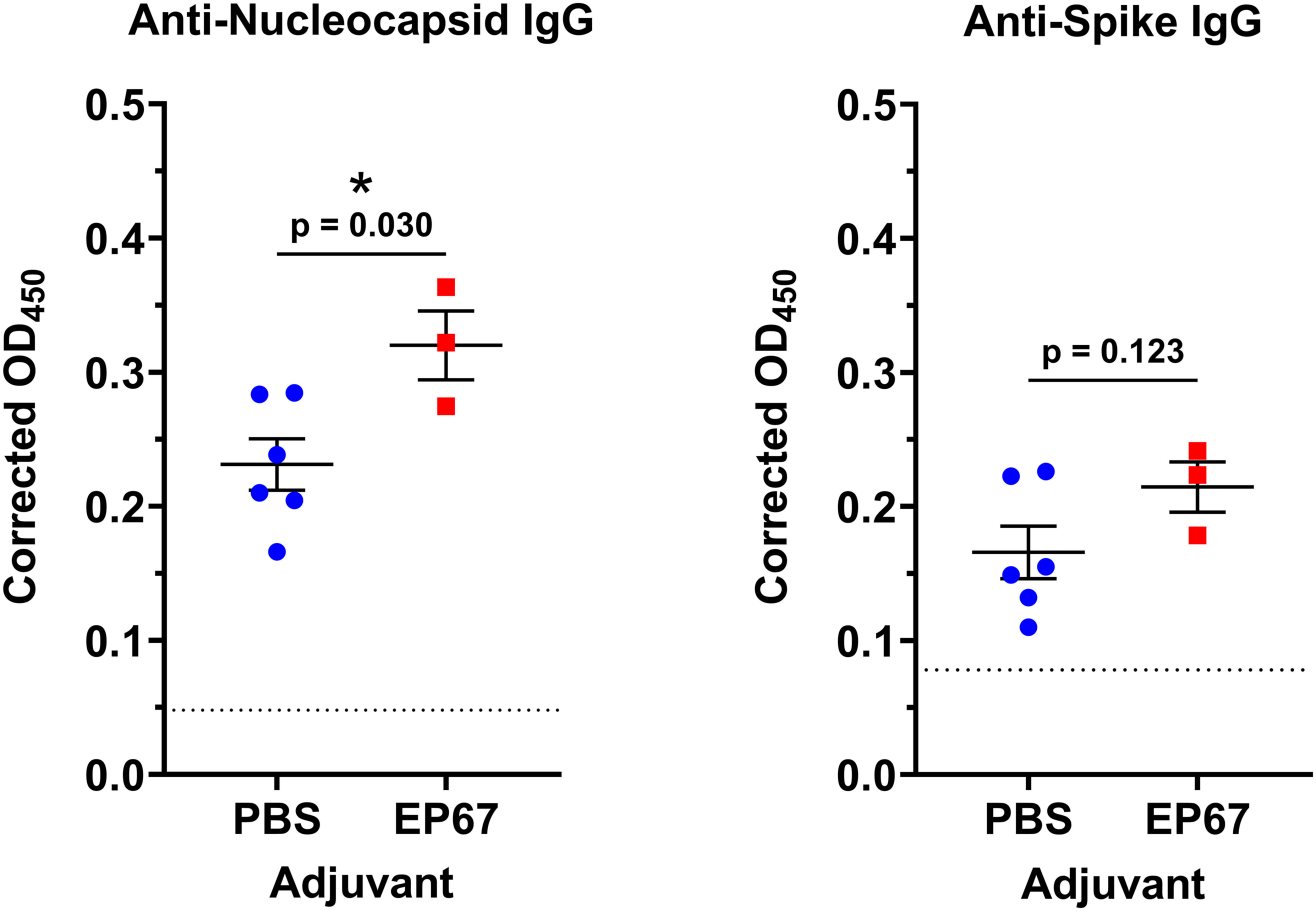
Mouse plasma anti-Nucleocapsid and anti-Spike IgG levels following intranasal immunizations with inactivated SARS-CoV-2 and adjuvant EP67. Mice received intranasal inoculations of either PBS vehicle (control; *n*=6) or 100 µg EP67 (*n*=3), co-administered with 2.7 x 10^3^ PFU of γ-irradiated SARS-CoV-2 Vero cell lysate on days 0 and 24. On day 42, K_2_EDTA plasma was obtained from submandibular bleeds, diluted 1:500, and an ELISA was performed to evaluate plasma IgG specific to SARS-CoV-2 Nucleocapsid (N) protein and Spike (S) glycoprotein. Mice receiving EP67-adjuvanted SARS-CoV-2 inactivated lysate demonstrated a significant increase in IgG levels toward Nucleocapsid protein, and an increase, though not significant, towards Spike glycoprotein, following immunizations compared to non-adjuvanted PBS vehicle controls. Each mouse plasma sample was tested in technical duplicates. A two-tailed unpaired t-test for statistical significance was performed using GraphPad Prism version 10.4.1. Colored symbols represent the average corrected OD_450_ of two technical replicates measurements for each mouse, and the corrected OD_450_ group mean ± SEM are displayed as long and short bars, respectively. The background signal of each assay is displayed as a horizontal dotted line. *p < 0.05.

To establish the capacity of EP67 to serve as a humoral immune adjuvant to SARS-CoV-2, mice were immunized with 2.7 x 10^3^ PFU of γ-irradiated (inactivated) SARS-CoV-2 Vero cell lysate coadministered with either 100 µg EP67 or PBS (vehicle control group). The irradiated viral lysate was leveraged in place of infectious live virus; however, this approach limits the utilization of the T_H_1 cell-mediated adaptive immune response, a known component of EP67 immune modulation (5). Nevertheless, following two immunizations spaced 24 days apart, mice receiving EP67-adjuvanted γ-irradiated SARS-CoV-2 lysate demonstrated significantly more viral nucleocapsid-specific IgG plasma antibodies compared to control mice receiving PBS and γ-irradiated SARS-CoV-2 lysate at Day 42 (p = 0.030). This suggests an apparent long-term, persistence in protection resulting from the immunizations at Days 0 and 24 (**Figure 1**).

**Figure 2:**
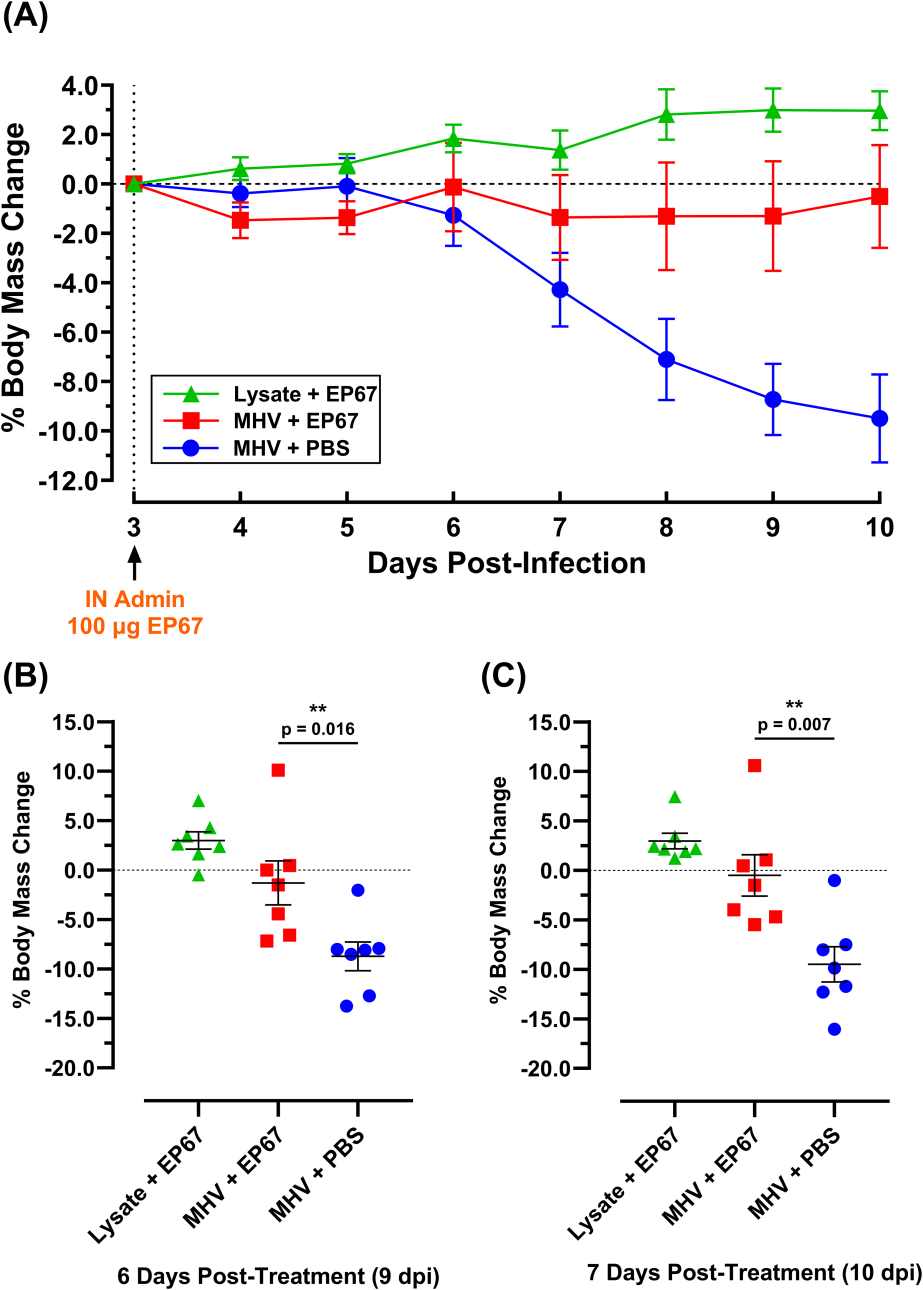
Body mass percent change of MHV-A59 infected and uninfected mice treated intranasally with PBS vehicle or immunostimulatory molecule EP67 three days post-infection. 7-week-old male C57BL/6J mice were infected intranasally (IN) with 17-Cl1 lysate containing 1.1 x 10^6^ PFU of betacoronavirus MHV-A59 or mock-infected with only 17Cl-1 cell lysate as the uninfected control. Each group had an *n*=10 mice up to day 5 post-infection (5 dpi) and *n*=7 mice for the remainder of the study. Three days after infection (3 dpi), mice were intranasally administered either 100 µg EP67 dissolved in PBS or PBS vehicle alone. **(A)** EP67-treated infected mice (red) experienced a return to health and maintained body mass, while untreated infected mice (blue) declined in health and body mass. Data presented as the group mean ± SEM. **(B)** At day 9 post-infection (“9 dpi”, 6 days post-treatment) and **(C)** day 10 post-infection (“10 dpi”, 7 days post-treatment), infected mice that received EP67 treatment (*n*=7) had significantly more body mass than infected, untreated (PBS vehicle control) mice (*n*=7). Two-tailed unpaired t-tests were performed using GraphPad Prism version 10.4.1 to determine statistical significance. Colored symbols represent individual mouse weights, and the group mean ± SEM are displayed as long and short bars, respectively. **p < 0.05.

To assess the ability of EP67 to serve as an effective treatment following the onset of illness from the related betacoronavirus Murine Hepatitis Virus (MHV), groups of 7-week-old male C57BL/6J mice were evaluated following infection. The C57BL/6 mouse strain has been previously used for infection with MHV strain A59 (MHV-A59), with disease progression correlated with observable illness and tissue pathology (12). Mouse body mass has been used extensively as a broad indicator of morbidity, and MHV infection in mice is known to significantly impact mouse health and weight loss, with higher doses proving lethal (12). In contrast, viral nucleic acid levels may persist in the tissues of animals with resolved infection for several days (16, 17). In the current study, each mouse received 1.1 x 10^6^ PFU MHV-A59, or 17Cl-1 uninfected host cell lysate, delivered intranasally on day 0. By day 3 post-infection (3 dpi), infected mice demonstrated a disease state with changes in behavior associated with viral infection, in agreement with previously published observations (12). To evaluate EP67 for therapeutic treatment efficacy following initial signs of infection, 100 µg of EP67 or PBS vehicle control was delivered intranasally on day 3 post-infection, and mice were monitored daily thereafter. Infected control mice receiving PBS declined in body mass, notably from day 6 post-infection onward, while EP67-treated mice maintained their body mass from the day of treatment throughout the end of the study and rapidly increased in health (**Figure 2**). Symptomatically, EP67-treated mice returned to health by day 3 post-treatment (corresponding to day 6 post-infection) and resembled the uninfected EP67-treated control mice. Statistical significance in the body mass percentage loss, relative to initiation of treatment on day 3 post-infection (3 dpi), was observed for infected EP67-treated versus infected PBS control mice 6 days post-treatment (9 dpi; **Figure 2B**) and 7 days post-treatment (10 dpi; **Figure 2C**).

**Figure 3:**
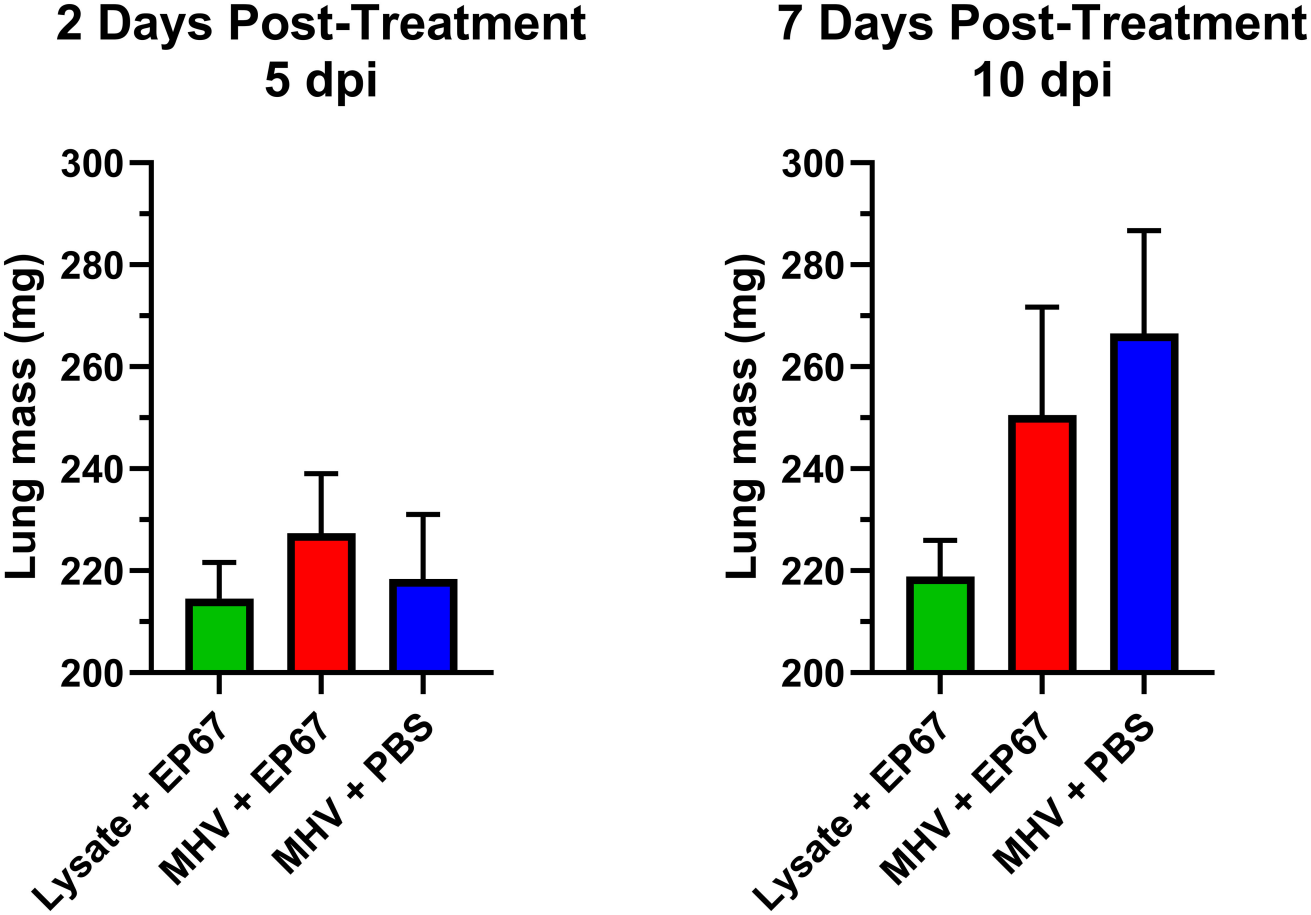
Lung masses of MHV-infected and uninfected mice treated intranasally with EP67 or PBS vehicle control. 7-week-old male C57BL/6J mice were infected intranasally (IN) with 17Cl-1 lysate containing 1.1 x 10^6^ PFU of betacoronavirus MHV-A59 or mock-infected with only 17Cl-1 cell lysate as the uninfected control. Three days post-infection (3 dpi), mice were intranasally administered either EP67 (100 µg) dissolved in PBS or PBS vehicle alone (infected untreated control). Lungs were dissected from euthanized mice at two days post-treatment (*n*=3 per group) and seven days post-treatment (*n*=4 per group) and immediately placed in 3 mL preservative at 4°C overnight, briefly blotted with a Kimwipe, and the mass of each mouse lung was measured using an analytical balance. No significant difference in lung mass of the MHV-infected mice treated with EP67 versus untreated PBS vehicle was detected, though lungs from EP67-treated infected mice did show a slight increase at two days post-treatment (5 dpi) and a modest decrease compared to the infected untreated PBS control mice at seven days post-treatment (10 dpi). As expected, uninfected mice mock-infected with 17Cl-1 cell lysate alone at day 0 (0 dpi), followed by EP67 administration at 3 dpi, maintained consistent lung masses at two and seven days post-treatment. Two-tailed unpaired t-tests for statistical significance were performed and figures showing each group’s mean lung mass ± SEM were generated using GraphPad Prism version 10.4.1.

Similar to SARS-CoV-2 infection in humans, intranasal infection of C57BL/6 mice by MHV strains (e.g. MHV-A59) is known to induce pathology by substantial recruitment of granulocytic cells to the lungs (12, 18, 19). The mean lung mass of EP67-treated infected mice (**Figure 3**) showed a slight increase at two days post-treatment (5 dpi) and a modest decrease compared to the infected untreated PBS control mice at seven days post-treatment (10 dpi). As expected, uninfected mice mock-infected with 17Cl-1 cell lysate alone at day 0 (0 dpi), followed by EP67 administration at 3 dpi, maintained consistent lung masses at two and seven days post-treatment, supporting the hypothesis that EP67 treatment did not aggravate the disease state further, but instead reduced disease severity within 24 hours based on behavior and measured weights. This suggests that in the context of this study, EP67, in contrast to its parent protein human C5a, does not appear to exacerbate host-mediated innate immune cascades (i.e. granulocyte effector functions) (6). Altogether, this evidence supports that EP67 treatment results in a significant decrease in morbidity following intranasal treatment at 3 days post-infection.

**Figure 4:**
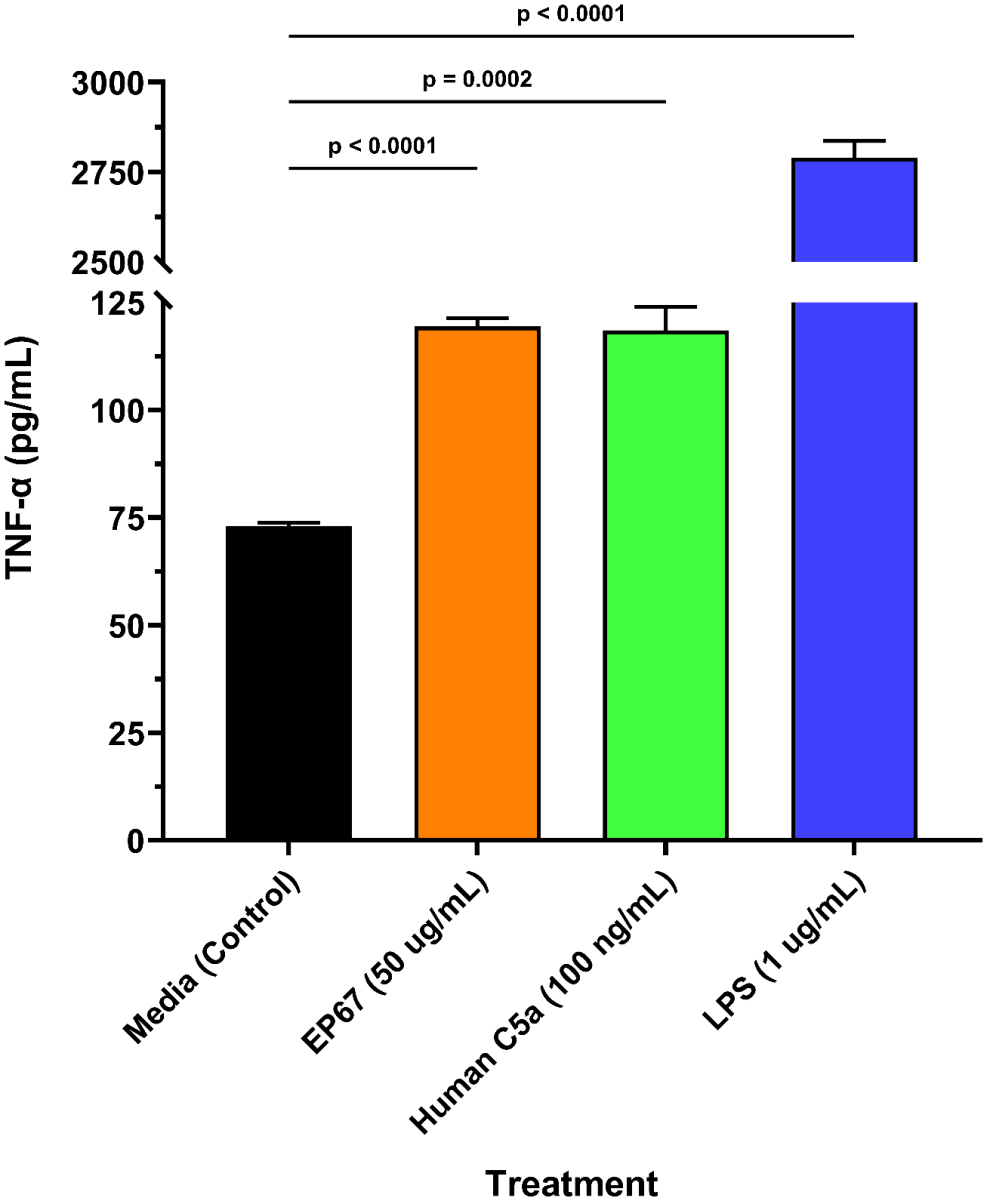
Murine macrophage release of TNF-α following EP67 exposure. Freshly prepared murine-derived RAW 264.7 macrophages were exposed to media only (control), human-derived C5a decapeptide EP67 (50 µg/mL), recombinant human parental complement molecule C5a (100 ng/mL), or lipopolysaccharide (LPS; 1 µg/mL) for 16 hours. Supernatants were collected to measure the release of TNF-α, a known anti-viral cytokine. Three wells per treatment (*n*=3) were assayed and each supernatant sample was tested in technical duplicates. A two-tailed unpaired t-test for statistical significance was performed using GraphPad Prism version 10.4.1. The group mean ± SEM of supernatant TNF-α levels are displayed.

The ability of EP67 to rapidly cause restored health following administration in symptomatic models of viral infection supports the view of this peptide as a potent and selective immunostimulant. It is theorized that EP67 invokes recruitment and activation of antigen-presenting cells (APCs) and enhances their antigen processing time and/or display levels, thereby driving the adaptive immune response a more rapid opportunity to develop B and T cell-mediated defenses against viral infection and propagation. Such bridging of the innate and adaptive immune responses is believed to underlie the observed rapid return to health. Previous studies with EP67 and its compositional predecessor EP54 support this hypothesis: murine dendritic cells internalized the peptide, upregulated selected activation-associated cytokines, and increased MHC expression compared to control (4). Similarly, murine splenic cells demonstrated marked increases in activation-related cytokines, and promoted enhanced cellular and humoral immune responses against the ovalbumin (OVA) antigen (5). In line with these previous findings, we show that EP67 is a significant (p < 0.0001) inducer of TNF-alpha (TNF-α) secretion from another APC: mouse RAW 264.7 macrophages (**Figure 4**). As seen in **Figure 4**, EP67 resulted in nearly the same magnitude as its parental protein, human C5a. The anti-viral capabilities of TNF-α are well-established, and patients who asymptomatically clear SARS-CoV-2 have been found with slightly higher expression compared to symptomatic patients (20). While high levels of TNF-α may contribute to deleterious inflammatory responses in some diseases, it often does so only in the context of other co-expressed inflammatory cytokines: such is the case with SARS-CoV-2, where TNF-α alone did not contribute to cell damage (21). Taken together, the EP67 molecule used here demonstrates an ability to directly activate murine antigen-presenting cells, and provide effective therapy for symptomatically infected mice, supporting its role as a selective immunostimulatory bio-mimetic of the parent C5a molecule.

## Conclusions

The SARS-CoV-2 global pandemic reiterated the urgent need for broad-spectrum antiviral agents, ideally with a capacity to treat individuals already infected. This brief study showed that the immunostimulant decapeptide, EP67, derived from human complement protein C5a, used in a single dose reduced disease severity of infection from mouse-native MHV, a betacoronavirus. EP67’s ability to return treated mice to a healthy state emphasizes its potential for safe application in the case of viral infections known to otherwise promote tissue destruction through elevated inflammatory responses. While used as a prophylactic adjuvant or as a treatment for symptomatic disease in this study, a greater understanding of EP67’s ideal therapeutic window will likely be an important element in future study designs (8). Finally, it would be valuable to advance the understanding on the molecular mechanisms responsible for this non-inflammatory immunostimulatory activity of EP67 and how it may bridge the innate and adaptive immune responses.

## Data Availability

The original contributions presented in the study are included in the article/supplementary material. Further inquiries can be directed to the corresponding author.
